# Improvement in IAPT outcomes over time: are they driven by changes in clinical practice?

**DOI:** 10.1017/S1754470X20000173

**Published:** 2020-06-09

**Authors:** Rob Saunders, John Cape, Judy Leibowitz, Elisa Aguirre, Renuka Jena, Mirko Cirkovic, Jon Wheatley, Nicole Main, Stephen Pilling, Joshua E.J. Buckman

**Affiliations:** 1 Centre for Outcomes Research and Effectiveness, Research Department of Clinical, Educational and Health Psychology, University College London, Gower Street, London, UK; 2 iCope – Camden and Islington Psychological Therapies Services, Camden & Islington NHS Foundation Trust, London, UK; 3 Redbridge Talking Therapies Service – North East London NHS Foundation Trust, London, UK; 4 Waltham Forest IAPT and Redbridge Talking Therapies Service – North East London NHS Foundation Trust, London, UK; 5 Talk Changes: City & Hackney IAPT Service, Homerton University Hospital NHS Foundation Trust, London, UK; 6 Let’s Talk IAPT – Barnet, Enfield & Haringey Psychological Therapies Service, Barnet, Enfield & Haringey Mental Health Trust, London, UK; 7 Camden & Islington NHS Foundation Trust, London, UK

**Keywords:** assessment, diagnosis, IAPT, psychological therapy, treatment outcome

## Abstract

Treatment outcomes across Improving Access to Psychological Therapies (IAPT) services in England have improved year-on-year, with the national average proportion of patients in recovery at the end of treatment now exceeding the 50% target. This is despite the number of referrals and numbers of treated patients also increasing year-on-year, suggesting that services have evolved local practices and treatment delivery to meet needs whilst improving performance. This study explores whether there have been changes in clinical practice with regard to: (1) the number of sessions and length of treatments; (2) the number of cancellations and non-attendance; and (3) the recording of problem descriptor information, and the association with treatment outcomes in IAPT. Routinely collected data from seven IAPT services involved in the North and Central East London (NCEL) IAPT Service Improvement and Research Network (SIRN) were brought together to form a dataset of nearly 88,000 patients who completed a course of IAPT treatment. Results showed that there was a slight increase in the average number of sessions, and decreases in the length of time in treatment, as well as decreases in both the number of non-attended appointments and the use of inappropriate problem descriptors. These findings highlight a number of areas where potentially small changes to clinical practice may have had positive effects on patient outcomes. The value of using IAPT data to inform service improvement evaluations is discussed.

**Key learning aims:**

(1) How changes to treatment-delivery factors are associated with IAPT patient outcomes.
(2) The link between clinical practice and potential service performance.
(3) How analysing routinely collected data can be used to inform service improvement.

## Introduction

The Improving Access to Psychological Therapies (IAPT) programme was developed in response to the rising burden of depression and anxiety disorders in England (Clark, 2011). Launched in 2008 by the Department of Health, IAPT aims to increase the availability of evidence-based (i.e. empirically supported) psychological interventions to patients in the National Health Service (NHS). In order to meet the growing demand, training of increasing numbers of clinicians is required, with an aim of over 10,500 new therapists to be trained by 2021 (Clark, 2018). Such is the perceived success of the programme that countries such as Australia (Cromarty *et al*., 2016) and Norway (Knapstad *et al.*, 2018) have adopted versions of the IAPT model for delivery in their own healthcare systems.

During the period from April 2018 to March 2019, over 1.09 million people were seen by IAPT services in England. Some received just an assessment and advice or signposting, whereas others (582,556 individuals) received a course of IAPT treatment (defined as two or more treatment sessions) (NHS Digital, 2019). This represents an 11.4% increase in referrals and a 5% increase in treated patients from the previous year. Despite this increased demand, IAPT services nationally have reported a year-on-year increase in the number of people recovering by the end of their treatment, with more than 50% reaching recovery across all services nationwide for the first time in early 2017 (Clark, 2018). Latest reports indicate that 52.1% of patients receiving a course of treatment recovered, up from 50.8% in the previous year (NHS Digital, 2019).

To achieve these levels of performance in the face of increasing pressures, IAPT services have had to evolve local practices in order to meet demands, yet little is known about how services have done this. One national evaluation of IAPT service performance between 2014 and 2016 (Clark *et al*., 2018) highlighted a number of factors that are associated with higher rates of reliable recovery and reliable improvement at the service level. These factors include the proportion of missed appointments across the service, the average number of treatment sessions delivered by the service, average waiting time between referral and entering treatment, and the index of multiple deprivation of the catchment area of the service, all of which were associated with reliable recovery and improvement. Importantly, the change at the service level in these factors from the first year analysed in that study (2014–2015) to the second year analysed (2015–2016) was also associated with changes in the proportion of patients achieving each outcome at the service level. So, for example, services that increased the average number of appointments or decreased the average waiting time between referral and starting treatment, from 2014–2015 to 2015–2016, reported higher proportions of patients achieving reliable recovery and reliable improvement at the end of treatment in 2015–2016 than they did in 2014–2015.

Analyses of cohorts of IAPT patients using individual patient data rather than aggregate data as used in the study noted above, have also found that the mean number of treatment sessions, the total length of time spent in treatment, and the number of treatment sessions that are cancelled, are all associated with treatment outcomes (Green *et al*., 2015; Gyani *et al*., 2013). Treatment non-attendance is an inefficient use of health service resources (Wells *et al*., 2013) and is associated with poorer outcomes from psychological interventions both in IAPT services and beyond (Schindler *et al*., 2013). Away from IAPT, research evidence has shown that increasing the frequency of cognitive behavioural therapy (CBT) sessions (delivering sessions more frequently) rather than the total number of sessions is associated with better treatment outcomes (Cuijpers *et al*., 2013; Herbert *et al*., 2004). Changes in the number of sessions, frequency of sessions, and attempts at reducing cancellations or non-attendance at treatment sessions could therefore have important implications for patient outcomes at the end of treatment. They could also potentially impact longer-term outcomes, as a failure to reach full recovery and experiencing residual symptoms at the end of treatment is one of the biggest predictors of relapse (Buckman *et al*., 2018b), and is associated with the need for further treatment from services up to a year after initially ending treatment (Ali *et al*., 2017; Buckman *et al*., 2018a).

One further factor that was highlighted from aggregate data at the service-level was the proportion of patients in the service who were given a ‘problem descriptor’ during their episode of care (Clark, 2018). IAPT services use the ‘problem descriptor’ variable (an ICD-10 code) to help match patients to National Institute for Health and Care Excellence (NICE) evidence-based treatments, which for CBT interventions includes the use of the appropriate disorder-specific CBT protocol (Clark, 2018). IAPT staff are trained to deliver the appropriate protocol for specific presenting problems, e.g. Clark and Wells (1995) model of CBT for social anxiety disorder, or Rapee and Heimberg (1997) for trauma-focused CBT for post-traumatic stress disorder (PTSD). Therefore, missing values on this variable for patients completing treatment might indicate the model used was not adequately matched to clinical needs. It has also been suggested that the prevalence of ‘mixed anxiety and depressive disorder’ (MADD) as a problem descriptor, indicating sub-threshold levels of depression and anxiety, is higher in some IAPT datasets than would be expected in epidemiological studies (Clark, 2011). Reviews of IAPT datasets, especially in the earlier years, have noted that patients coded with MADD had baseline symptom severity scores above threshold levels (Gyani *et al*., 2013), which would suggest MADD was probably an inappropriate problem descriptor for these cases.

IAPT services are mandated to collect routine outcome measures at each session, which results in high quality data that can be used to inform service improvement. With 98.5% completion of pre- and post-treatment outcome measures (Clark, 2018), IAPT datasets have great potential to highlight potential areas of clinical practice that could be adapted to improve patient care and service performance. The North and Central East London IAPT Service Improvement and Research Network (NCEL IAPT SIRN) was established in order to use routinely collected IAPT data from all individual patients that have been seen in the local services, for the purpose of sharing best practice and improving the care these services provide. The aim of this paper is to analyse changes in local practices and the corresponding change in individual patient outcomes reported by NCEL IAPT services. Specifically, this analysis will focus on annual changes in a number of variables that are mentioned in the IAPT manual (a guide for commissioners, managers and IAPT clinicians to support the expansion and development of local IAPT services) as being important to consider for improving recovery rates (National Collaborating Centre for Mental Health, 2018). These variables are: (1) the number of treatment sessions and length of treatment episodes; (2) the number of cancelled or non-attended appointments; and (3) the use of problem descriptors and the change in local service outcomes.

## Method

### Participants

**Figure 1.**
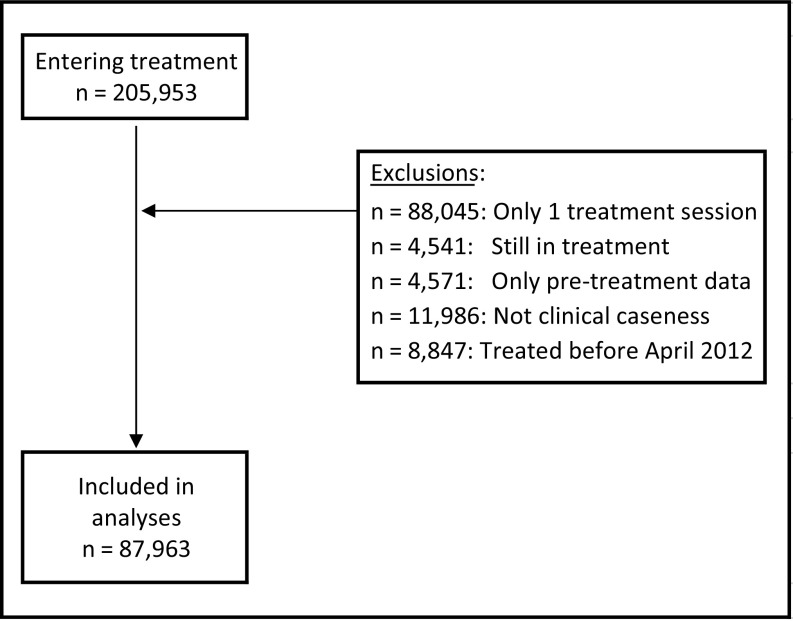
Patient flow diagram.

The NCEL IAPT SIRN dataset includes information provided by seven local IAPT services. The dataset used for the current analysis includes routinely collected data from all patients who had a course of IAPT treatment (two or more treatment sessions) in NCEL IAPT services, were in caseness at the start of treatment (i.e. they had symptoms of either depression or anxiety, suggestive of a probable diagnosis of some depressive or anxiety disorder; see ‘Measures and outcomes’ section below for details) and completed pre- and post-treatment outcome measures. Furthermore, it was decided to use data from the 2012–2013 financial year (April to March) onwards as not all services were established by this time, and this allowed for a comparison of all services across all years. A total of *n* = 87,963 patients met inclusion criteria and provided data for the current analyses. See Fig. 1 for the flow of patients into this study.

### Measures and outcomes

The NCEL IAPT SIRN dataset includes a number of measures related to the process of treatment that have been identified in previous analyses as having a potential impact on IAPT service performance and patient outcomes (Clark *et al*., 2018; Green *et al*., 2015). Three sets of these variables will be explored in the current analysis, and were chosen as they are amenable to change in service practice. These are defined below:

(1) The mean number of treatment sessions per patient and the average duration (in weeks) between the first and last treatment appointments that each patient attended, each financial year.
(2) The mean number of appointments for each patient that were cancelled by the IAPT clinician and the mean number of did-not-attends (DNAs) per patient for each financial year.
(3) The proportion of patients with a missing problem descriptor and the proportion of patients coded as mixed anxiety and depressive disorder (MADD) in each financial year.

Two IAPT-defined patient outcomes were considered in the current study, both of which are used in national IAPT reporting (NHS Digital, 2019). The first outcome, ‘recovery’, is defined in IAPT as moving from scoring above caseness for either depression or anxiety at the start of treatment to scoring below caseness on measures of both depression and anxiety symptoms at the end of treatment. The second outcome, ‘reliable improvement’, is defined as a reduction in symptom scores above the error of measurement for the depression and anxiety measures used.

Depression symptom severity was measured using the Patient Health Questionnaire 9-item version (PHQ-9; Kroenke *et al*., 2001), where scores of 10 or above indicate caseness for depression, and a reduction of 6 or more points on the scale indicates reliable improvement in depression symptoms (NHS Digital, 2016, 2017).

The Generalized Anxiety Disorder Scale 7-item (GAD-7; Spitzer *et al*., 2006) is the main measure of anxiety symptoms used in IAPT services. Caseness is defined as scores of 8 or above, and a reduction of 4 or more points indicates reliable improvement.

Alternative measures of anxiety symptoms are used in IAPT when a specific anxiety problem descriptor is identified, such as the Social Phobia Inventory (Connor *et al*., 2000) as the appropriate measure of social anxiety disorder. For details about the thresholds and cut-offs for each of these additional measures, please see the IAPT national reports (e.g. NHS Digital, 2016).

### Plan of analysis

Data were extracted on all patients in the NCEL IAPT SIRN dataset who met inclusion criteria, and were initially split into financial years. The recovery and reliable improvement outcomes were calculated for each patient. The mean number of treatment sessions, duration of treatment, number of cancellations and DNAs, the proportion of missing and MADD problem descriptors and percentage of patients reaching recovery and reliable improvement were derived for each financial year. The percentage of patients reaching the two outcomes in each financial year was presented graphically against: (1) the mean number of treatment sessions and the average duration of treatment, (2) the mean number of cancellations and DNAs and (3) the proportion of missing diagnoses and proportion MADD diagnoses (in patients who were scoring above caseness) per financial year.

Further analysis was then conducted to explore whether each of the treatment-delivery factors defined above were associated with recovery or reliable improvement using logistic regression analyses. Analyses were performed at the individual patient level, and the number of treatment sessions, duration of treatment, number of DNAs, number of cancellations by the service, whether the patient’s problem descriptor was missing and whether the patient’s problem descriptor was recorded as MADD were all entered both univariate and multiple logistic regressions models. Multiple logistic regressions were run to include all six treatment-delivery factors alongside baseline depression and anxiety symptom severity, as these are known to predict recovery and reliable improvement (Saunders *et al*., 2016), as well as the service code to control for potential differences between services. All analyses were conducted in stata15 (StataCorp, 2017).

## Results

### Descriptive analyses

#### Number of sessions and duration of treatment

**Figure 2.**
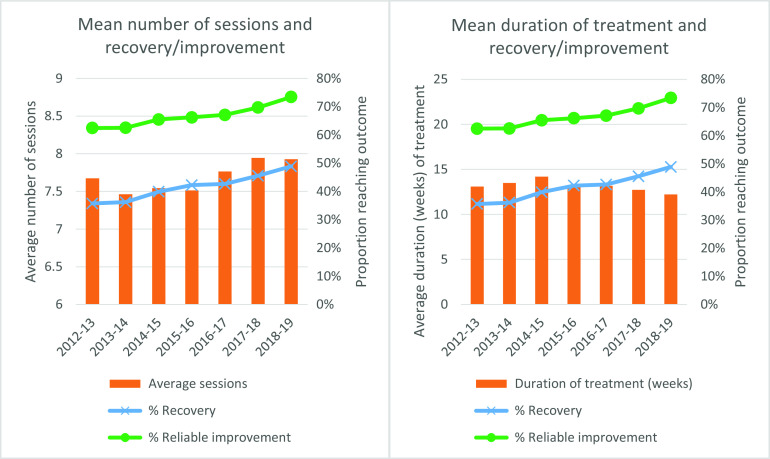
Average number of treatment sessions per episode and average duration of treatment, by financial year.

The average number of treatment sessions per year for patients who completed a course of treatment (two or more sessions) across the NCEL services is presented in the left-hand panel of Fig. 2, with the yearly percentage of patients reaching recovery and reliable improvement superimposed. At a glance, the graphs suggest that there has been a slight increase in average sessions over time, and this has coincided with a yearly improvement in outcomes. Further analysis indicates that the reduction in the number of treatment sessions per episode was statistically significant (at *p* < 0.05) between the years 2012–2013 and 2013–2014, and that the increases between 2015–2016 and 2016–2017, and between 2016–2017 and 2017–2018 were also statistically significant (see Supplementary material, Appendix, Table A1).

The right-hand panel of Fig. 2 presents the yearly change in the average length of time of IAPT episodes (see Supplementary material, Appendix, Table A1). Following a peak in weeks in treatment in the 2014–2015 financial year, there has since been a drop in the average duration of treatment in weeks, which has coincided with increases in the proportions of patients achieving recovery and reliable improvement across the services. The initial increase in the weeks in treatment between 2012–2013 and 2013–2014 was found to be statistically significant, as were the decreases between 2014–2015 and 2015–2016, between 2016–2017 and 2017–2018, and between 2017–2018 and 2018–2019. Taken together, the graphs appear to indicate that despite the average number of sessions increasing year by year, the mean time in treatment has decreased, which might suggest that treatments are being provided more frequently, with less time elapsing between treatment sessions.

#### Number of appointment cancellations/non-attendance

**Figure 3.**
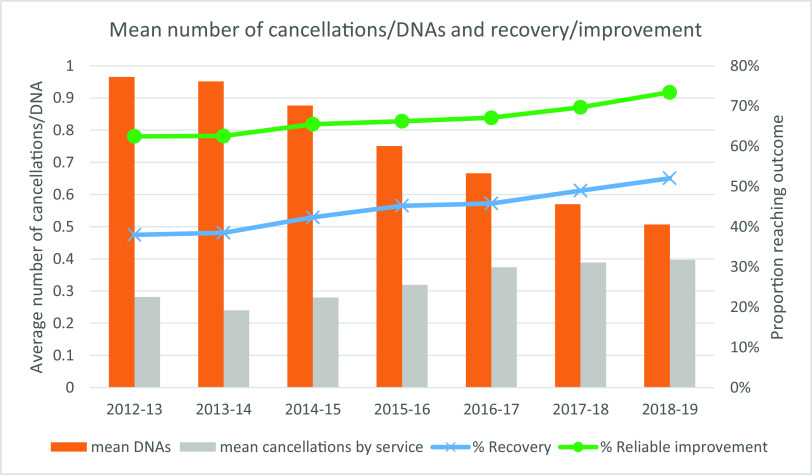
Mean number of cancellations and DNAs per treatment episode, by financial year.

Figure 3 presents the yearly mean number of DNAs and cancellations (by the service) per patient over the course of their treatment. There has been a clear reduction in the mean number of DNAs per person over time, reductions which were found to be statistically significant between each year from 2013–2014 (see Supplementary material, Appendix, Table A2), suggesting that patient attendance rates have improved, which has coincided with an increasing proportion of patients reaching recovery and reliable improvement per year. The mean number of service-related cancellations per episode has fluctuated from a mean of 0.28 cancellations in 2012–2013 up to 0.39 in 2018–2019, indicating an increase of nearly 40%. Tests indicated that the number of cancellations significantly decreased between 2012–2013 and 2013–2014 before it significantly increased year by year between 2013–2014 and 2016–2017 (see Supplementary material, Appendix, Table A2).

#### Problem descriptor use

**Figure 4.**
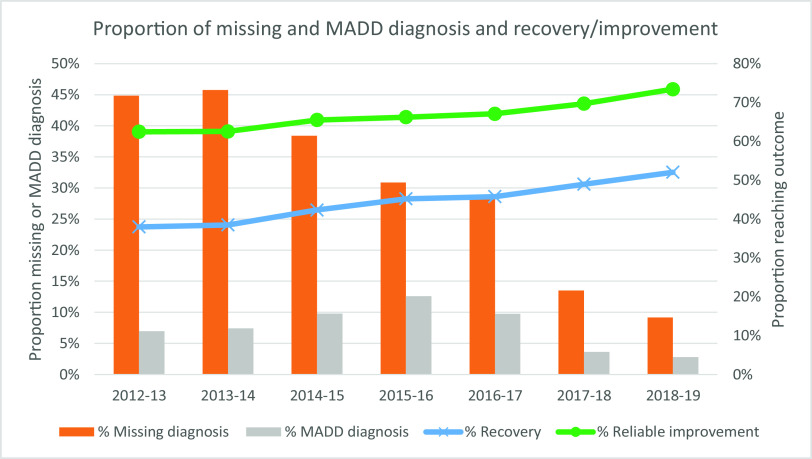
Proportion of patients with a diagnosis of MADD or without a diagnosis recorded, by financial year.

The proportion of patients either missing information about their problem descriptor, or who were given MADD as their problem descriptor, despite scoring above caseness on the PHQ-9 or the GAD-7 [or appropriate Anxiety Disorder Specific Measure (ADSM)] are presented in Fig. 4. There has been a clear reduction in missing problem descriptor information over the last 7 years, with around 45% missing in 2012–2013, down to less than 10% missing in 2018–2019. These decreases were significantly different between each year from 2013–2014 (see Supplementary material, Appendix, Table A3). The proportion of MADD identification has also decreased, with a peak in the 2015–2016 years, which has decreased down to less than 3%. The year by year increases between 2013–2014 and 2015–2016 were significant, as were the year by year decreases between 2015–2016 and 2018–2019 (see Supplementary material, Appendix, Table A3).

#### Logistic regression analyses

The association of treatment-delivery factors and baseline symptom severity with both recovery and reliable improvement in individual patients was then explored in the next set of analyses. Results are presented in Table 1 and the top panel shows that each of the treatment-delivery factors was significantly associated with recovery in univariable models (left-hand columns). Having more sessions of treatment was associated with a higher likelihood of recovery in univariable models, whereas more DNAs and cancellations, as well as having a missing or MADD problem descriptor were all associated with a lower likelihood of recovery. There was a very small significant effect of a longer duration of treatment being associated with greater odds of recovery, and higher baseline severity scores on both the PHQ-9 and GAD-7 were associated with lower odds of recovery. In multivariable models, controlling for all of the treatment-delivery variables, all variables were still significantly associated with recovery except having a missing problem descriptor. The direction of the odds ratio for duration of treatment switched so that having more days in treatment was associated with a lower likelihood of recovery when controlling for other variables (whereas longer duration was associated with a higher likelihood of recovery in the univariable model). This is probably due to the number of sessions already being controlled for, and indicates that delivering the same number of sessions more frequently improves the odds of recovery.

**Table 1.** Logistic regression analyses comparing odds of recovery and reliable improvement for each treatment-delivery factor

Recovery						
Variable	Unadjusted odds ratio	95% CI	*p*-value	Adjusted odds ratio*	95% CI	*p*-value
Baseline PHQ-9 score	0.891	(0.888–0.893)	<0.001	0.913	(0.91–0.916)	<0.001
Baseline GAD-7 score	0.886	(0.883–0.889)	<0.001	0.935	(0.931–0.939)	<0.001
Number of sessions	1.048	(1.045–1.051)	<0.001	1.066	(1.061–1.071)	<0.001
Duration of treatment	1.006	(1.005–1.007)	<0.001	0.995	(0.994–0.997)	<0.001
Count of DNAs	0.741	(0.731–0.751)	<0.001	0.779	(0.768–0.79)	<0.001
Count of cancellations	0.964	(0.944–0.983)	<0.001	0.925	(0.937–0.946)	<0.001
Problem descriptor = missing	0.954	(0.927–0.983)	0.002	1.003	(0.966–1.041)	0.883
Problem descriptor = MADD	0.843	(0.802–0.886)	<0.001	0.939	(0.887–0.994)	0.03

Reliable improvement						
Variable	Unadjusted odds ratio	95% CI	*p*-value	Adjusted odds ratio*	95% CI	*p*-value
Baseline PHQ-9 score	0.993	(0.991–0.996)	<0.001	0.972	(0.969–0.975)	<0.001
Baseline GAD-7 score	1.034	(1.031–1.037)	<0.001	1.059	(1.054–1.063)	<0.001
Number of sessions	1.086	(1.082–1.089)	<0.001	1.090	(1.084–1.095)	<0.001
Duration of treatment	1.014	(1.013–1.015)	<0.001	0.993	(0.992–0.995)	<0.001
Count of DNAs	0.846	(0.836–0.856)	<0.001	0.850	(0.839–0.86)	<0.001
Count of cancellations	1.039	(1.017–1.061)	<0.001	0.915	(0.894–0.937)	<0.001
Problem descriptor = missing	0.847	(0.822–0.874)	<0.001	0.911	(0.877–0.946)	<0.001
Problem descriptor = MADD	0.925	(0.878–0.974)	0.003	0.884	(0.835–0.936)	<0.001

* Adjusted for all treatment-delivery variables, baseline severity and service.

The results of the logistic regression analyses for reliable improvement are presented in the lower panel of Table 1. The univariable models presented in the left-hand columns show that all variables were significantly associated with reliable improvement. More sessions and longer duration of treatment were individually associated with increased odds of reliable improvement, whereas more DNAs and having missing or MADD as the patient’s problem descriptor were associated with decreased odds of reliable improvement. Patients with more cancelled appointments appeared to have greater odds of reliable improvement in the univariable model, but this was not the case when adjusting for other factors, suggesting that the initial effect was most likely due to more cancellations being associated with more sessions overall (having more sessions was significantly correlated with increased number of cancellations: *r* = 0.22, *p* < 0.001). As expected, the direction of the effect for cancellations and treatment days is reversed in the multivariable models (right-hand columns). Results show that more sessions increase the odds of improvement whilst controlling for the other variables, and that more weeks in treatment, more DNAs and cancellations and having either MADD or missing problem descriptor information was associated with decreased odds of reliable improvement. Findings also showed that higher baseline PHQ-9 scores were associated with lower odds of reliable improvement, in both univariate and adjusted models, but higher GAD-7 scores were associated with increased odds of reliable improvement. This finding might be due to some baseline dependency in the outcome, as those scoring higher have more available points on the symptom scores to drop and therefore meet criteria for reliable improvement (Saunders *et al*., 2019b). However, it is interesting that this only exists for GAD-7 scores and not for PHQ-9 scores, suggesting a differential impact of baseline depression and anxiety scores on outcomes.

## Discussion

The presented analysis of individual patient data provided by seven IAPT services from the last seven financial years has shown a number of potential treatment-delivery factors that are associated with the likelihood of patients achieving recovery and reliable improvement at the end of their IAPT treatment. The factors considered in the current analysis are all mentioned in the IAPT manual (National Collaborating Centre for Mental Health, 2018) in relation to improving service recovery rates. The mean number of treatment sessions has gradually increased over time, whereas the average duration from the start to finish of treatment has more recently decreased, both of which may be associated with the improved service level outcomes observed. The mean number of DNAs has steadily dropped, whereas cancellations by the services have slightly increased. There have been large decreases in the proportion of patients missing a problem descriptor code and the proportion of patients who were in caseness pre-treatment but had been incorrectly coded as having MADD. All these factors were associated with recovery and reliable improvement in univariable models. When controlling for all of the other factors, having more treatment sessions, fewer weeks in treatment, fewer DNAs and cancellations, and not having a MADD problem descriptor were all associated with increased odds of recovery and reliable improvement. Whereas the findings around treatment sessions, cancellations, DNAs and problem descriptor completion replicate previous findings (Clark *et al*., 2018; Gyani *et al*., 2013), previous analyses have not considered the duration of IAPT treatment on outcomes.

The number of sessions and the duration of treatment were associated with outcomes here and have previously been found to be associated with psychological treatment outcomes both in IAPT (Green *et al*., 2015) and other settings (Cuijpers *et al*., 2013; Erekson *et al*., 2015). The univariable regression models showed that increases in both the number of sessions and days in treatment were associated with improved outcomes, yet in the multivariate models it was found that increasing weeks in treatment actually decreased the odds of positive treatment outcomes. This is because the multivariable models controlled for the effect of the number of sessions, and therefore when number of sessions was held constant results showed that increasing the duration in treatment was associated with poorer outcomes. The finding resonates with the expected delivery of cognitive behavioural interventions, where it is expected that the first four sessions are delivered within two weeks, moving to weekly then fortnightly sessions (Beck, 2011), whereas the time pressures in IAPT services, as in most routine treatment services, might mean that twice-weekly CBT sessions are not possible. However, the results might indicate a change in clinical practice that the treatment appointment scheduling should consider the time between treatment sessions. Although the number of treatment sessions was highlighted by Clark *et al*. (2018) as being important in predicting outcome, the duration of treatment was not explored in the previous analysis. However, the current findings suggest that treatment duration may be an additional treatment-delivery factor that could be considered in improving IAPT patient outcomes by clinicians, managers and commissioners.

The reduction in year by year DNAs might be explained by the better use/availability of patient liaison capabilities from the commonly used electronic patient record systems in IAPT which are now able to send automatic reminders to patients (through email and text messaging) about upcoming appointments and to ask them to complete their routine outcome measures. The further use of technology for patient engagement purposes such as online therapy or computerised guided self-help materials could result in continued improvements in service engagement and patient outcomes. In addition, it is likely that this is something that varies between services quite considerably, whereby there may be local attendance or DNA policies which dictate practice in one service but not another. For example, some services might operate policies around the maximum number of DNAs any service user is able to have before they are automatically discharged back to the care of their GP. It is also common practice in some services for service users and their clinicians to sign an informal ‘treatment contract’ in which the number of sessions and use of between-session activities (sometimes considered to be ‘homework’) are laid out. Where this is true it would be very unusual to have patients with many cancellations or DNAs, but where these practices or policies are not in place, we might expect that the number of cancellations and DNAs would fluctuate to a much greater extent.

The current analysis showed that a trend in better problem descriptor completion (less missing and less MADD) was associated with the increase in outcomes. By selecting an appropriate problem descriptor, it is expected that the IAPT clinician has considered the presenting problem(s) and therefore matched the clinical issue to appropriate evidence-based NICE guidelines and CBT protocol (Clark, 2018). Equally, it may be that clinicians were able to identify the appropriate clinical problem, but they forgot or were otherwise unable to record the ICD-10 code on the system. This may further explain why patients who had a problem descriptor recorded as MADD despite scoring above caseness on either/both depression and anxiety symptom measures were at higher risk of poor outcomes, as it is more likely that the clinical problem and appropriate disorder-specific CBT protocol was not as well considered in treatment planning. Overall the results highlight the importance of correctly identifying the presenting problem and selecting the appropriate evidence-based treatment protocol for the identified disorder.

The findings of this study replicate some of the previous national service level analyses by Clark *et al.* (2018), which found that an increase in the average number of appointments, frequency of missed appointments and the proportion of patients with missing problem descriptor information were all associated with treatment outcomes at the level of services as a whole. Unlike the previous study, the current study has used individual patient data from IAPT services greatly increasing the power of the analyses allowing the inclusion of data from nearly 88,000 patients, instead of using pooled statistics from just over 200 services. More importantly, by examining associations at the level of each IAPT patient rather than associations aggregated across services we can be more confident that the results are not biased by the ‘ecological fallacy’, i.e. when trying to interpret the findings of the group level analyses for individual patients, ignoring heterogeneity within the groups that were the subject of analysis. One potential drawback of using individual patient data is the reduced availability of system or process variables, which have been shown to be associated with outcomes. For example, the study by Clark *et al*. included service-level deprivation and the proportion of patients entering treatment who had a full course of treatment (i.e. did not receive just one treatment session only), which were found to be associated with outcomes. However, these factors could not be considered in the current analysis as they are not specific to individuals, and instead they are specific to relatively small geographical areas in which a number of individuals might live.

It is important to add that the findings of both the current analyses and those of Clark *et al*. (2018) reinforce the recommendations of the IAPT manual in relation to ‘improving recovery’ in IAPT services (National Collaborating Centre for Mental Health, 2018). Although the IAPT manual is focused on ‘recovery’, which is perceived as the major outcome of IAPT services, the current analysis has shown how changes in these treatment-delivery factors have had a similar impact on reliable improvement in patient symptoms as well. A further important finding was that higher baseline GAD-7 scores were associated with a higher likelihood of achieving reliable improvement by the end of IAPT treatment. This suggests that even those with very high scores on the GAD-7 pre-treatment can benefit from IAPT treatment. The factors highlighted in both the manual and the current analysis suggest that small changes to clinical practice can have positive benefits to outcomes in IAPT services. However, such changes should remain within the scope of national guidelines on evidenced-based treatment for common mental health disorders seen within IAPT. The use of large individual patient data datasets such as the NCEL IAPT SIRN dataset can provide a means with which to feed back the impact of specific changes on clinical practice on patient outcomes, and provide a method of sharing best practice.

### Changes to practice made by services

Clinical leads from the NCEL IAPT SIRN services were approached about changes to local practices, policies or strategies that had been adopted in their services. A range of responses were received. Some involved improving relationships with GPs to improve the appropriateness of referrals, and seeing patients within GP surgeries to make attendance easier for the patients, thus reducing DNAs (Foustanos *et al*., 2018). Others involved removing arbitrary caps on the maximum numbers of clinical sessions offered, particularly at high intensity, and changing attendance policies so that there were differences in how patients were notified about expectations of attendance and the consequences of non-attendance. In some services this took the form of changing the way messages were given to patients about attendance in letters and text message reminders prior to a session taking place, with information on the impact of DNAs and last-minute cancellations for other patients waiting to be seen being emphasised in those messages. A small group of services piloted (and in some instances then used more widely) the use of internet-based messenger systems and other services piloted the use of internet-based video calls to conduct sessions with patients that were less able to attend face-to-face; this was said by clinical leads to have reduced the number of DNAs and cancellations of such sessions. Other services introduced changes to their contracts with external agencies providing interpreters for sessions to be held with people who did not speak English or who preferred to have an interpreter to work with in their preferred language. The services also held regular training sessions with staff on working with interpreters, and anecdotally the staff in those services suggested this may have led to a reduction in the number of cancellations by the service.

Several initiatives focused on improving staff’s understanding and accuracy in the routine recording of presenting problems. Such initiatives included whole staff training sessions either with senior clinicians within the services or with internationally recognised experts in the treatment of particular disorders, bringing questions about presenting problems to every supervision session, sending emails from managers to remind staff that their client’s had not had a recorded presenting problem, and drop-in diagnosis advice clinics with senior staff. In addition, one group of services got the provider of the electronic patient record system they use to change the appearance of the ‘patient details’ section of the patient record in order to include reminders to their staff about the use of presenting problems, with a particular focus on ensuring appropriate use of MADD. These changes appear to have had a considerable impact when looking at the decreases in missing and inappropriate problem descriptor use over the last few years.

A number of services made a point of focusing on patient recovery and reliable improvement outcomes, introducing training and workshops with staff to share best practice, and in two services they introduced 6-monthly one-to-one meetings with a line manager (‘recovery consultations’) which included an in-depth look at a number of cases where patients the staff member had worked with did recover and a number of cases where their patients did not recover, in order to highlight commonalities and discrepancies. These meetings were also set up so that they finished with an agreed set of objectives and progress against them would be reviewed at the subsequent meeting 6 months later. The objectives might include attending additional training for working with particular disorders, a change in the use or frequency of supervision, partnering with another (usually more experienced) staff member to answer questions about coding and recording of data on the electronic patient record system, or in some cases agreeing to more regular oversight of the quality of routinely collected data by a given staff member with reminders of best-practice in order to support them to improve their recovery rates (Foustanos, 2018). Other services also focused on the quality of routinely collected data, ensuring that their staff were completing all necessary data fields and that they recognised the importance of collecting outcome data to the functioning of the services.

Lastly, a number of services indicated that an increased focus on staff wellbeing, including appointing a wellbeing lead within the service, had improved staff and service dynamics, which they believe had resulted in improved performance and had been highlighted by staff as improving working practices in annual staff surveys (Saines, 2018). Although the direct impact of such changes could not be measured in the current study, an interesting piece of further research would be to explore strategies of improving staff wellbeing and exploring how this can positively impact patient care.

### Limitations

Although this study benefits from a large sample of patients, with data spanning 7 years to assess change in both clinical practice and outcomes, there are a number of limitations to the current analysis. Firstly, the dataset includes individual patient data from seven IAPT services, and although the trends in outcome seem to mirror those nationally, the treatment-delivery factors explored may operate differently in other IAPT services outside of the NCEL SIRN. Exploring the impact of these factors on change in performance in other services would be of value to assess the wider impact. A further limitation is that the current analysis is focused on two outcomes (recovery and reliable improvement) only and ignores other potentially important patient outcomes that may have also changed over time. Recovery and reliable improvement were chosen as the outcomes for this analysis due to their importance to IAPT services and local clinical practice, but other more patient-focused outcomes could be considered in further evaluations. The current analysis focused on patients who completed a course of treatment (2+ sessions) and were caseness at the start of treatment, as standard for national evaluations of IAPT services. However, this ignores patients receiving only one treatment session and those with less severe presentations to services, and there may be important changes in the number of patients receiving only one session or who were below caseness across time that might further influence changes in patient outcome, as suggested by Clark *et al*. (2018).

The analysis was also limited with regard to the variables considered, and other factors not considered may have influenced findings. For example, the use of specific types of interventions or sub-types of treatments may have changed within services, potentially increasing the use of computer-based interventions, or more/less group delivered treatments, which could be associated with changes in outcomes, and these could not be explored in the current study. This is in part because IAPT treatments delivered in services frequently offer a range of treatment types within an episode of care, which is common in other types of routine treatment services and is why IAPT national reports use the ‘last therapy type’ for reporting (NHS Digital, 2016). However, this does not reflect the variation in therapy type that may have occurred before the last sessions and therefore more detailed analysis would be required in order to use therapy type information.

There has been much research into the role of potential patient-related factors that can predict treatment outcomes (Green *et al*., 2015; Gyani *et al*., 2013; Saunders *et al*., 2016; Saunders *et al*., 2019a), and potentially there may have been year-by-year changes in the presentation of patients to services which may have resulted in changes to service performance. For example, previous analyses have identified distinct profiles of patients attending IAPT services with significant differences in outcomes observed between these profiles (Saunders *et al*., 2016). If the distribution of profiles has varied over time, with ‘less complex’ profiles increasing in prevalence then it is possible that a change in patient profiles may be associated with improved performance. This was not considered in this analysis of treatment-delivery factors, as these patient-related factors cannot be influenced by clinical practice, and instead this analysis is concerned with changes to clinical practice that may be associated with outcomes. Analysis of annual mean PHQ-9 and GAD-7 scores suggests a small decrease in scores (PHQ-9: mean score was 15.9 in 2012–2013 and 15.2 in 2018–2019; GAD-7: mean score was 14.3 in 2012–2013 and 14.0 in 2018–2019), with further analyses indicating some statistically significant decreases between years in the mean baseline PHQ-9 score, and both significant increases and decreases in the mean baseline GAD-7 scores (see Supplementary material, Appendix, Table A3). However, multivariate regression models controlling for baseline severity did not alter the findings, indicating that the impact of treatment-delivery factors identified in the current analysis was independent of initial patient severity.

### Conclusion

Taken together, the results indicate that the delivery of more sessions, provided at more frequent intervals, reduced cancellations and the correct identification of a patient’s presenting problem are associated with better outcomes in IAPT services, and that the year-by-year changes in these factors is associated with improving outcomes being reported nationally. A number of these factors are already suggested in the IAPT manual, and this analysis supports the consideration of these factors in service planning. The analysis benefits from a large sample of individual patient data, and builds on previous analyses of the IAPT national data. The use of aggregated datasets from IAPT services such as the one from the NCEL SIRN presented in the current analysis could be used to identify areas of clinical practice that are associated with improved patient outcomes and potentially identify aspects of service delivery that could be adjusted to optimise care. The sharing of this information could support best practice across services.

## Supplementary material

For supplementary material accompanying this paper visit https://doi.org/10.1017/S1754470X20000173. click here to view supplementary material

## Key practice points

(1) Routinely collected information about how treatment is delivered can be used to highlight areas of improvement.
(2) More treatment sessions, delivered in a more condensed period of time, was associated with better outcomes.
(3) The correct identification of specific IAPT-related presenting problems and reduced non-attendance was linked to a higher likelihood of recovery and reliable improvement.
