# Supplementary material for: Improvement in IAPT outcomes over time: are they driven by changes in clinical practice?
Source: Cogn Behav Therap. 2020 Jun 9;13:e16. doi: 10.1017/S1754470X20000173 (PMC7872157; doi:10.1017/S1754470X20000173)
Supplement: Supplementary file 1 [file S1754470X20000173sup001.docx]

Appendix to:

**Improvement in IAPT outcomes over time: are they driven by changes in clinical practice?**

Supporting data of year by year change in treatment-delivery factors.

**Table A1. Number of treatment sessions and weeks of treatment per year.**

|  | Weeks in treatment | | Count treatment sessions | |  | Count LI sessions | Count HI sessions |
| --- | --- | --- | --- | --- | --- | --- | --- |
| Financial Year | Mean(sd) | p-value* | Mean(sd) | p-value* |  | Mean(sd) | Mean(sd) |
| 2012-13 | 13.09 (14.74) |  | 7.67 (5.31) |  |  | 2.44 (2.84) | 5.03 (5.65) |
|  |  | 0.054 |  | 0.043 |  |  |  |
| 2013-14 | 13.5 (14.15) |  | 7.46 (4.97) |  |  | 3.06 (2.96) | 4.19 (5.2) |
|  |  | <0.001 |  | 0.192 |  |  |  |
| 2014-15 | 14.19 (14.59) |  | 7.54 (5.01) |  |  | 3.2 (2.77) | 4.19 (5.31) |
|  |  | <0.001 |  | 0.627 |  |  |  |
| 2015-16 | 13.17 (14.79) |  | 7.51 (4.96) |  |  | 3.1 (2.75) | 4.37 (5.33) |
|  |  | 0.985 |  | <0.001 |  |  |  |
| 2016-17 | 13.17 (14.47) |  | 7.76 (4.96) |  |  | 2.92 (2.82) | 4.82 (5.41) |
|  |  | 0.010 |  | 0.002 |  |  |  |
| 2017-18 | 12.74 (12.88) |  | 7.94 (5.02) |  |  | 2.8 (2.76) | 5.11 (5.55) |
|  |  | 0.001 |  | 0.780 |  |  |  |
| 2018-19 | 12.21 (11.82) |  | 7.93 (4.84) |  |  | 3.16 (2.84) | 4.75 (5.51) |

Notes: * p-values from independent samples t-tests between years.

Table A1 presents the mean weeks in treatment and number of treatment sessions for each year included in the analysis. The mean weeks in treatment significantly increased (at p<0.05) between the years 2013-14 and 2014-15, before it significant decreased between 2014-15 and 2015-16, 2016-17 and 2017-18, and 2017-18 and 2018-19. The mean number of treatment sessions per episode significantly decreased between the years 2012-13 and 2013-14, and then increased between 2015-16 and 2016-17 as well as between 2016-17 and 2017-18. The mean number of low intensity (LI) and high intensity (HI) treatments by year is also presented. These appear to have fluctuated over time, so the general increase in number of sessions from 2013-14 to 2018-19 does not seem to be due to changes in the allocation of one intensity of treatment only.

**Table A2. DNAs, Cancellations, missing diagnoses and MADD diagnoses per year.**

|  | DNA | | Cancellations Service | | Missing diagnosis | | MADD diagnosis | |
| --- | --- | --- | --- | --- | --- | --- | --- | --- |
| Financial Year | Mean(sd) | p-value* | Mean(sd) | p-value* | % | p-value‡ | % | p-value‡ |
| 2012-13 | 0.97 (1.43) |  | 0.28 (0.61) |  | 44.83% |  | 6.98% |  |
|  |  | 0.495 |  | <0.001 |  | 0.199 |  | 0.222 |
| 2013-14 | 0.95 (1.53) |  | 0.24 (0.55) |  | 45.74% |  | 7.43% |  |
|  |  | <0.001 |  | <0.001 |  | <0.001 |  | <0.001 |
| 2014-15 | 0.88 (1.37) |  | 0.28 (0.61) |  | 38.39% |  | 9.79% |  |
|  |  | <0.001 |  | <0.001 |  | <0.001 |  | <0.001 |
| 2015-16 | 0.75 (1.14) |  | 0.32 (0.65) |  | 30.88% |  | 12.59% |  |
|  |  | <0.001 |  | <0.001 |  | <0.001 |  | <0.001 |
| 2016-17 | 0.67 (1.05) |  | 0.37 (0.7) |  | 28.20% |  | 9.76% |  |
|  |  | <0.001 |  | 0.093 |  | <0.001 |  | <0.001 |
| 2017-18 | 0.57 (0.96) |  | 0.39 (0.73) |  | 13.51% |  | 3.64% |  |
|  |  | <0.001 |  | 0.379 |  | <0.001 |  | <0.001 |
| 2018-19 | 0.51 (0.91) |  | 0.4 (0.76) |  | 9.16% |  | 2.79% |  |

Notes: * p-values from independent samples t-tests between years.

‡ p-values from chi-square tests between years.

The annual mean ‘did not attends’ (DNAs) and cancellations by the service per treatment episode, as well as the annual proportion of episodes with missing diagnosis information and diagnoses of mixed anxiety and depressive disorder (MADD) in patients meeting caseness, is presented in Table A2. The number of DNAs per episode significantly decreased year by year from 2013-14. The number of cancellations significantly decreased between 2012-13 and 2013-14 before it significantly increased year by year between 2013-14 and 2016-17.

The likelihood of missing diagnosis information significantly decreased every year from 2013-14, from 46% missing in 2013-14 down to 9% in 2018-19. The number of incorrect MADD diagnoses significantly increased between 2013-14 and 2014-15, and 2014-15 and 2015-16, before it significantly decreased year by year between 2015-16 and 2018-19.

**Table A3. Mean PHQ-9 and GAD-7 scores per year.**

|  | PHQ-9 | | GAD-7 | |
| --- | --- | --- | --- | --- |
| Financial Year | Mean(sd) | p-value* | Mean(sd) | p-value* |
| 2012-13 | 15.88 (5.58) |  | 14.33 (4.37) |  |
|  |  | 0.685 |  | 0.673 |
| 2013-14 | 15.91 (5.65) |  | 14.3 (4.42) |  |
|  |  | <0.001 |  | 0.387 |
| 2014-15 | 15.64 (5.65) |  | 14.25 (4.39) |  |
|  |  | 0.021 |  | <0.001 |
| 2015-16 | 15.49 (5.6) |  | 14.01 (4.45) |  |
|  |  | 0.930 |  | 0.074 |
| 2016-17 | 15.48 (5.6) |  | 14.1 (4.45) |  |
|  |  | <0.001 |  | 0.002 |
| 2017-18 | 15.16 (5.6) |  | 13.94 (4.47) |  |
|  |  | 0.489 |  | 0.004 |
| 2018-19 | 15.21 (5.49) |  | 14 (4.42) |  |

Notes: * p-values from independent samples t-tests between years.

The mean baseline PHQ-9 and GAD-7 scores per year are presented in Table A3. Independent samples t-tests indicate that the mean PHQ-9 significantly decreased between the years 2013-14 and 2014-15, 2014-15 and 2015-16, as well as 2016-17 and 2017-18. Mean baseline GAD-7 scores significant decreased between 2014-15 and 2015-16, and 2016-17 and 2017-18, whilst they significantly increased between 2017-18 and 2018-19.
